# Association between statewide financial incentive programs and COVID-19 vaccination rates

**DOI:** 10.1371/journal.pone.0263425

**Published:** 2022-03-30

**Authors:** Harsha Thirumurthy, Katherine L. Milkman, Kevin G. Volpp, Alison M. Buttenheim, Devin G. Pope

**Affiliations:** 1 Department of Medical Ethics and Health Policy, Perelman School of Medicine, University of Pennsylvania, Philadelphia, Pennsylvania, United States of America; 2 Department of Operations, Information and Decisions, The Wharton School, University of Pennsylvania, Philadelphia, Pennsylvania, United States of America; 3 Department of Family and Community Health, School of Nursing, University of Pennsylvania, Philadelphia, Pennsylvania, United States of America; 4 Booth School of Business, University of Chicago, Chicago, Illinois, United States of America; Carnegie Mellon Univeristy, UNITED STATES

## Abstract

To promote COVID-19 vaccination, many states in the US introduced financial incentives ranging from small, guaranteed rewards to lotteries that give vaccinated individuals a chance to win large prizes. There is limited evidence on the effectiveness of these programs and conflicting evidence from survey experiments and studies of individual states’ lotteries. To assess the effectiveness of COVID-19 vaccination incentive programs, we combined information on statewide incentive programs in the US with data on daily vaccine doses administered in each state. Leveraging variation across states in the daily availability of incentives, our difference-in-differences analyses showed that statewide programs were not associated with a significant change in vaccination rates. Furthermore, there was no significant difference in vaccination trends between states with and without incentives in any of the 14 days before or after incentives were introduced. Heterogeneity analyses indicated that neither lotteries nor guaranteed rewards were associated with significant change in vaccination rates.

## Background

Despite widespread availability of COVID-19 vaccines, about half of Americans are not fully vaccinated and vaccination rates have declined since their peak in April 2021. To encourage vaccination, many states introduced incentive programs ranging from guaranteed rewards (e.g., small rewards like free beverages or larger rewards like gift cards of up to $100) to lotteries in which vaccinated individuals had a chance to win $1 million or more. Lotteries in particular have been widely used because of evidence that people tend to overweight small probabilities and thus respond to large jackpots more than they would to small cash payments [1]. However, it is unclear whether such incentive programs are effective at increasing COVID vaccination rates given the strong disinterest many of the unvaccinated may have in being vaccinated.

Existing studies of incentives for COVID-19 vaccination offer conflicting accounts of their effectiveness. A survey experiment in Germany suggests guaranteed rewards of about $25 or higher can significantly increase vaccine uptake [2]. In contrast, evaluations of the first vaccine lottery in Ohio have yielded mixed results [3, 4]. The relatively early introduction of incentives in Ohio may also limit the generalizability of results from there. One other study examining statewide incentive programs introduced prior to early-June 2021 concluded that lottery incentives were effective in 10 of 12 states that were studied [5]. However, methodological limitations stemming from cross-sectional-analysis confounds and unadjusted correlation in errors in daily vaccination rates within a state warrant further evaluation of these programs. Heterogeneity in the success of statewide incentive programs remains underexplored as well. Lotteries and guaranteed rewards may vary in their effectiveness. Given substantial political partisanship in COVID-19 prevention policies [6, 7], conservative and liberal states may also have varying success in promoting vaccination with incentives.

We study the effectiveness of statewide incentive programs for COVID-19 vaccination using detailed information on 24 statewide incentive programs and data on daily vaccine doses administered in each state. We also examine heterogeneity in program effectiveness based on key characteristics of incentive programs and the states where they were introduced.

## Methods

We compiled information on statewide incentive programs from the National Governors’ Association [8] and Google News. For each program, we recorded the start date (when vaccinations were first incentivized), end date, and incentive type (lottery or guaranteed reward). For states with multiple programs, we recorded the dates for the program with the highest expected value per resident. We calculated the number of vaccine doses administered daily per 100,000 individuals in each state with data from the US Centers for Disease Control and Prevention [9], focusing on the period April 1, 2021 (before the first statewide incentive program began) to July 29, 2021. We also classified states based on whether they voted for the Republican or Democratic Party candidate in the 2020 Presidential Election.

For each state-date combination, we created an indicator variable reflecting an active incentive program. Leveraging variation across states in the daily presence of incentives, we used difference-in-differences regressions to examine the association between incentive programs and vaccination rates. Specifically, to determine this association during the entire period when incentives were active, we estimated the following model: *Vaccinations_jt_* = *Incentive_jt_*+*θ_j_*+*γ_t_*, where *j* indexes the state and *t* indexes the date. *Incentive*_*jt*_ is our indicator variable for an active incentive program in state *j* on date *t*, and *θ_j_* and *γ_t_* are fixed effects for each state and day, respectively, in order to adjust for time-invariant differences across states in vaccination rates and for national trends over time. The standard errors we report are clustered to account for arbitrary correlation of error terms at the state level.

To test for heterogeneity in the effectiveness of incentive programs, we performed subgroup analyses that separately examined effects of lotteries vs. guaranteed rewards, incentive programs introduced early vs. late (based on the median date when incentive programs were introduced in our sample), and incentive programs in Republican- vs. Democratic-leaning states.

In contrast to the difference-in-differences analyses that combined data from all states that had incentive programs, we also used a synthetic control approach that examined the impact of each state’s incentive program one at a time relative to a synthetic control comprised of the “control group” of states that never had incentive programs. The synthetic control was defined on the basis of the daily vaccination rate in control group states in the 1 day before an incentive program was introduced as well as the 8 days before the program was introduced.

In additional analyses, we assessed whether responsiveness to incentives may have been greatest in the weeks immediately after incentives were introduced. In these analyses we also tested whether pre-program time trends were similar between states with and without incentive programs, a key assumption of the difference-in-differences model. Specifically, we defined indicator variables for each of the 14 days before and after incentive programs were introduced and estimated the following model that compared daily vaccination rates between states with and without incentive programs in the two 14-day periods, while again clustering standard errors at the state level: ${Vaccinations}_{jt}={\sum_{t=-14}^{14}Incentive}_{jt}+\theta_{j}+\gamma_{t}$. This model allowed us to look at vaccination trends leading up to the date when incentives were introduced and the subsequent effect of incentives on vaccination uptake.

## Results

Twenty-four states introduced vaccination incentive programs during the study period (Table 1). The median (interquartile range) percent of the population that was fully vaccinated when incentives began was 43.8% (39.3%-47.2%). Among these states, vaccination rates declined from a daily average of 486/100,000 individuals in the 14 days pre-incentives to a daily average of 351/100,000 individuals in the 14 days post-incentives. This reflected a national trend, as daily vaccination rates also declined in the 26 states without statewide incentive programs during comparable 14-day periods (from 351/100,000 to 272/100,000 individuals vaccinated daily). Difference-in-differences analysis showed that overall, incentive programs were associated with a non-significant relative decline in daily vaccination rates of 8.9/100,000 individuals (p = 0.75) during the period when incentives were deployed (Table 2, Column 1). The 95% confidence interval for this main effect suggests we can rule out that the incentive programs increased daily vaccinations by 45/100,000 (a ~10% increase in daily vaccination rates given the average daily vaccinations in our dataset). In the Appendix, we show the evolution of vaccination rates in the period before and after incentives were introduced in each of the 24 states with incentive programs as well as that state’s unique synthetic control (S1 Appendix). While our main results in Table 2 show that incentive programs did not increase vaccination rates, the results from the synthetic control analyses allow readers to visually inspect each state’s incentive program individually for suggestive evidence that incentives may have been effective in a few of the states.

**Table 1:** Summary of analyzed statewide incentive programs for COVID-19 vaccination and trends in daily vaccination rates.

						Daily vaccinations per 100k, mean		
State	Start date	End date	Eligibility, Minimum age	Incentive type	Description of primary incentive programs	14 days before	14 days after	Difference
Connecticut	19-May	31-May	All, 12	Guaranteed	Free drink at restaurants	837	583	-254
New Jersey	19-May	4-Jul	All, 12	Guaranteed	Free annual state park pass	735	554	-181
Minnesota	27-May	30-Jun	New, 12	Guaranteed	$25 Ticket/Pass	623	317	-306
Ohio	13-May	23-Jun	All, 12	Lottery	Five $1 Million prizes	548	394	-154
Maryland	20-May	3-Jul	All, 18	Lottery	One $400,000 prize + Daily drawings	819	549	-270
New York	20-May	11-Jun	New, 18	Lottery	One $5 Million prize	696	556	-140
Oregon	21-May	27-Jun	All, 12	Lottery	One $1 Million prize	840	622	-217
Colorado	25-May	30-Jun	All, 12	Lottery	Five $1 Million prizes	637	382	-255
Delaware	25-May	29-Jun	All, 12	Lottery	One $302,000 prize	538	438	-100
New Mexico	1-Jun	6-Aug	All, 18	Lottery	One $5 Million prize	451	241	-210
Washington	3-Jun	11-Jul	All, 12	Lottery	One $1 Million prize	675	446	-230
Hawaii	4-Jun	31-Aug	All, 18	Lottery	$34,150 total prizes	422	274	-147
Kentucky	4-Jun	25-Aug	All, 12	Lottery	Three $1 Million prizes	297	314	18
North Carolina	10-Jun	1-Aug	All, 12	Lottery	Four $1 Million prizes	180	169	-12
Massachusetts	15-Jun	19-Aug	All, 12	Lottery	Five $1 Million prizes	452	347	-105
Maine	16-Jun	3-Jul	All, 12	Lottery	One $896,809 prize	431	218	-213
Illinois	17-Jun	19-Aug	All, 12	Lottery	Three $1 Million prizes	346	360	14
Louisiana	17-Jun	31-Jul	All, 12	Lottery	One $1 Million prize	218	157	-61
Nevada	17-Jun	26-Aug	All, 12	Lottery	One $1 Million prize	305	293	-13
Michigan	1-Jul	30-Jul	All, 12	Lottery	One $2 Million prize + Daily drawings	225	123	-102
Missouri	21-Jul	6-Oct	All, 12	Lottery	900 $10,000 prizes	156	240	85
West Virginia	20-May	1-Aug	All, 12	Both	$100 gift card/US treasury bond + One $1.6 Million prize	289	233	-56
Arkansas	26-May		New, 12	Both	$20 Game/Fish Certificate, One $1 Million prize	290	201	-89
California	27-May	18-Jul	New, 12	Both	$50 gift card + Ten $1.5 Million prizes	662	413	-249
All 24 states with incentive programs	n/a	n/a	n/a	n/a	n/a	486	351	-135
All 26 states without incentive programs	n/a	n/a	n/a	n/a	n/a	351	272	-79

Eligibility indicates whether all vaccinated individuals or newly vaccinated were eligible for incentives. Program descriptions focus on incentives that were typically offered for individuals aged ≥18 years, as individuals aged 12–17 years typically received other incentives such as scholarship funds. Among the 26 states without statewide incentive programs, the average daily vaccine doses administered per 100,000 individuals are calculated over the 14-day periods before (and after) the start dates of incentives in the 24 states with statewide incentive programs.

**Table 2:** Association between statewide incentive programs and vaccination rates, difference-in-difference analysis.

	Dependent Variable: Daily Vaccinations in State, Per 100,000 Individuals						
		Incentive type		Incentive begin date		Political partisanship	
	All States	Lottery Incentives Only	Included Guaranteed Incentives	Early (May 27 or Before)	Late (After May 27)	Republican-Voting States	Democrat-Voting States
	(1)	(2)	(3)	(4)	(5)	(6)	(7)
State Incentive Program in Progress	-8.9	-22.7	1.7	0.2	-53.1	56.8*	-51.1*
	(27.5)	(32.9)	(43.8)	(27.7)	(49.9)	(30.6)	(29.9)
State Fixed Effects	X	X	X	X	X	X	X
Date Fixed Effects	X	X	X	X	X	X	X
Observations	5,880	5,171	3,760	4,468	4,463	3,879	5,052
Number of States in Treatment Group	24	18	6	12	12	7	17
Number of States in Comparison Group	26	26	26	26	26	26	26
Mean of Dependent Variable	429	423	401	420	407	375	443

All subgroup analyses included states that never introduced incentives. * Significant at 10%.

In subgroup analyses, neither lottery incentives (in 18 states) nor the provision of guaranteed incentives (in 6 states) had significant effects on vaccination rates (Table 2, Columns 2 and 3). As columns 4 and 5 show, incentives also did not have a significant effect on vaccination rates in states that began offering incentives before or after the median date when statewide incentive programs began (May 27, 2021). Finally, Columns 6 and 7 of Table 2 provide suggestive evidence that incentives were (marginally) effective in promoting vaccination in states with Republican-leaning electorates, which had considerably lower vaccination rates than Democratic-leaning states. In Republican-leaning states, incentive programs were associated with an increase in daily vaccination rates of 56.8/100,000 individuals (p = 0.073). In contrast, incentives were associated with a decline in daily vaccination rates of 51.1/100,000 individuals (p = 0.095) in states with Democratic-leaning electorates.

**Fig 1:**
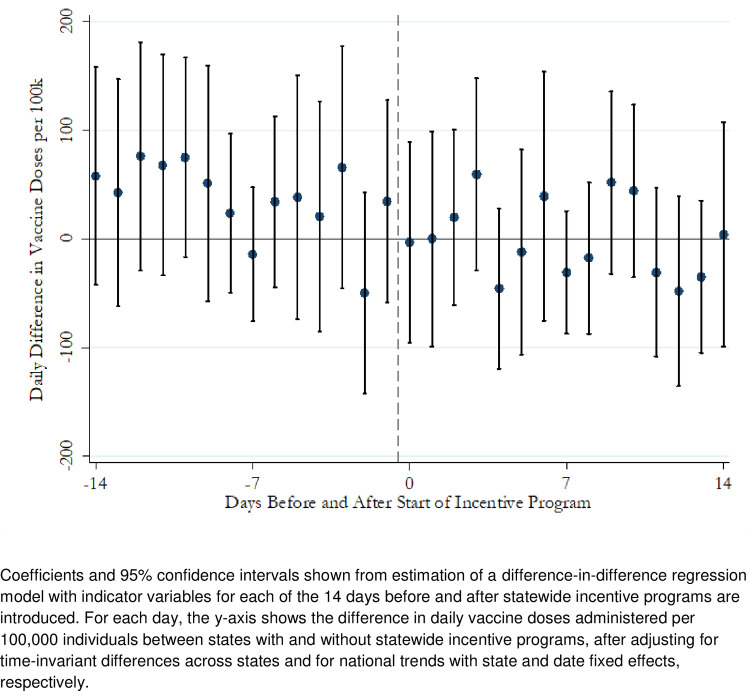
Difference-in-differences analysis of daily vaccination rates in states with and without incentive programs, 14 days before and after start of incentive programs. Coefficients and 95% confidence intervals shown from estimation of a difference-in-difference regression model with indicator variables for each of the 14 days before and after statewide incentive programs are introduced. For each day, the y-axis shows the difference in daily vaccine doses administered per 100,000 individuals between states with and without statewide incentive programs, after adjusting for time-invariant differences across states and for national trends with state and date fixed effects, respectively.

Comparing vaccination rates in the 14 days *before* incentives were introduced, we confirmed that states with and without incentive programs had similar trends in vaccination rates (Fig 1)–a finding that supports the parallel trends assumption in our difference-in-differences analyses. Each point in Fig 1 shows the difference between states with and without incentive programs in the days leading up to and after the date when incentives were introduced. In the 14 days before incentives, there did not appear to be a sharp increase or decrease in the difference in vaccination rates. This was confirmed with an F-test that tested the joint significance of the coefficients. A joint test of the coefficients for the 7 days before incentives found that the coefficients were not significantly different (F(7,49) = 1.21; p = 0.31). Expanding to the 14 days before incentives, we found a marginally significant difference (F(14, 49) = 1.98; p = 0.04), that was driven by slight differences between states with and without incentives in the 7–14 days before incentives were introduced. Furthermore, there was no significant difference in vaccination trends between states with and without incentives in any of the 14 days *after* incentives were introduced. The latter result provides additional confirmation that the main difference-in-difference results do not mask short-term increases in vaccination rates after incentives were launched.

## Discussion

Lotteries and other incentives offered by 24 states were not associated with a significant change in COVID-19 vaccination rates. Adjusting for national trends in vaccination rates and correlation in daily vaccination rates within states, this study goes beyond existing studies of statewide programs that have focused on individual states or may not have adjusted for confounding factors. Confidence intervals for our analyses indicate we had insufficient statistical power to detect small effects of incentives, but that increases of greater than 10% in daily vaccination rates can be ruled out. Our findings are also consistent with recent evaluations of city-wide lottery incentives like those offered in Philadelphia [10]. Many factors likely explain our findings. With about 40% of individuals already fully vaccinated when incentives were introduced—and significant resistance to vaccination among many of the unvaccinated—small rewards (e.g. $5-$50) or low-probability lotteries may have been insufficiently persuasive to unvaccinated individuals. Incentives have been effective in other contexts [11], but their impact may be attenuated among those whose vaccine intentions are shaped by misinformation or distrust. In certain contexts in which incentives have been effective at promoting healthy behavior (such as smoking cessation on weight loss), individuals who were offered incentives typically had an underlying desire to change their behavior whereas the desire to get vaccinated may be minimal for many who are now being offered incentives. Low awareness of incentive programs may also reduce effectiveness. A limitation of the study is that employer or local government incentive programs that we did not observe may have dampened the effects of statewide programs. Overall, our findings suggest that more substantial incentives or mandates may be necessary to raise vaccination rates.

## Supporting information

S1 Appendix (PDF)

S1 Data (DTA)
