# Supplementary material for: Association between statewide financial incentive programs and COVID-19 vaccination rates
Source: PLoS One. 2022 Mar 30;17(3):e0263425. doi: 10.1371/journal.pone.0263425 (PMC8966995; doi:10.1371/journal.pone.0263425)
Supplement: S1 Appendix — (PDF) [file pone.0263425.s001.pdf]

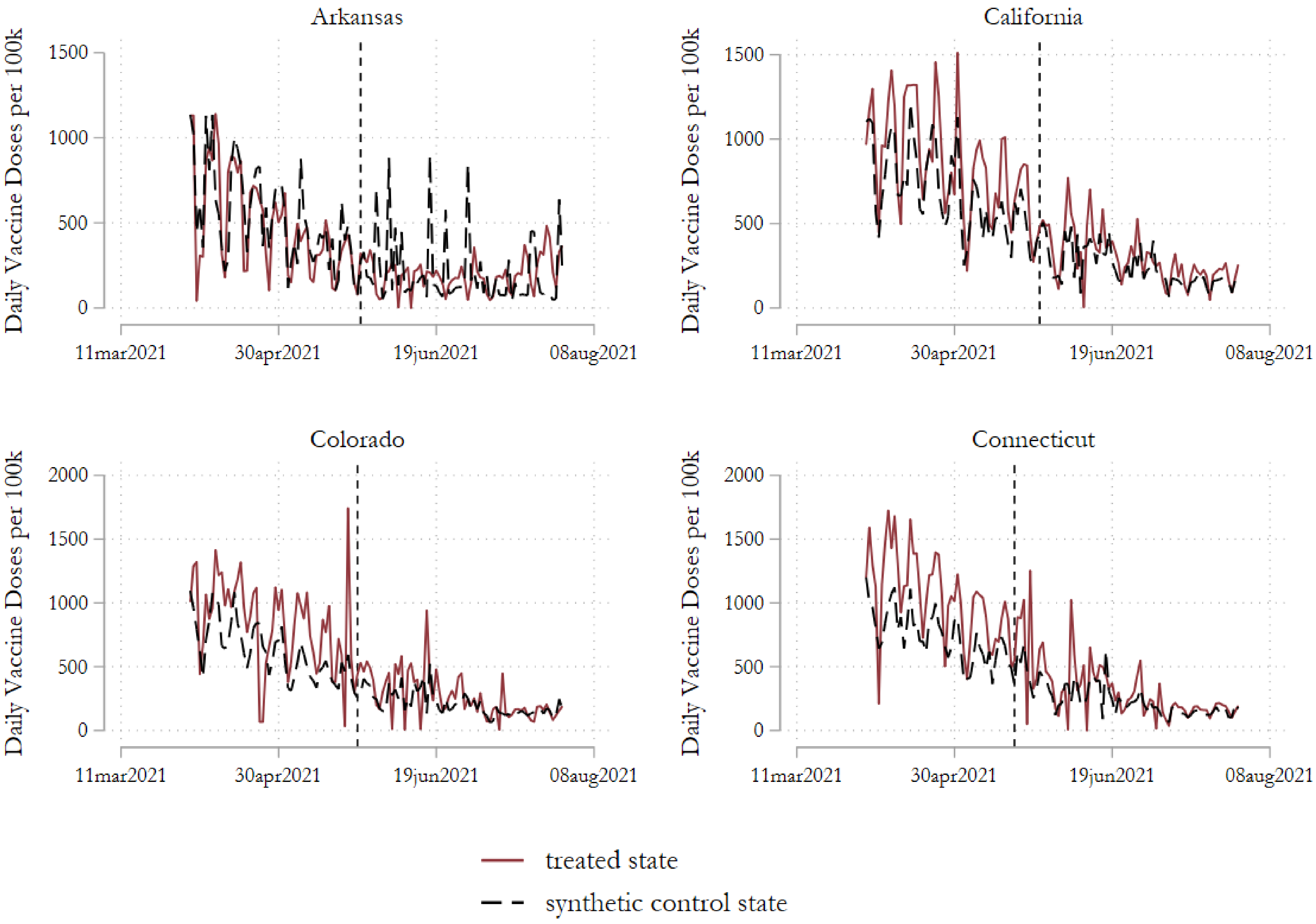

Arkansas
California
Colorado
Connecticut
Daily Vaccine Doses per 100k
1500
1000
500
0
2000
11mar2021
30apr2021
19jun2021
08aug2021
treated state
synthetic control state

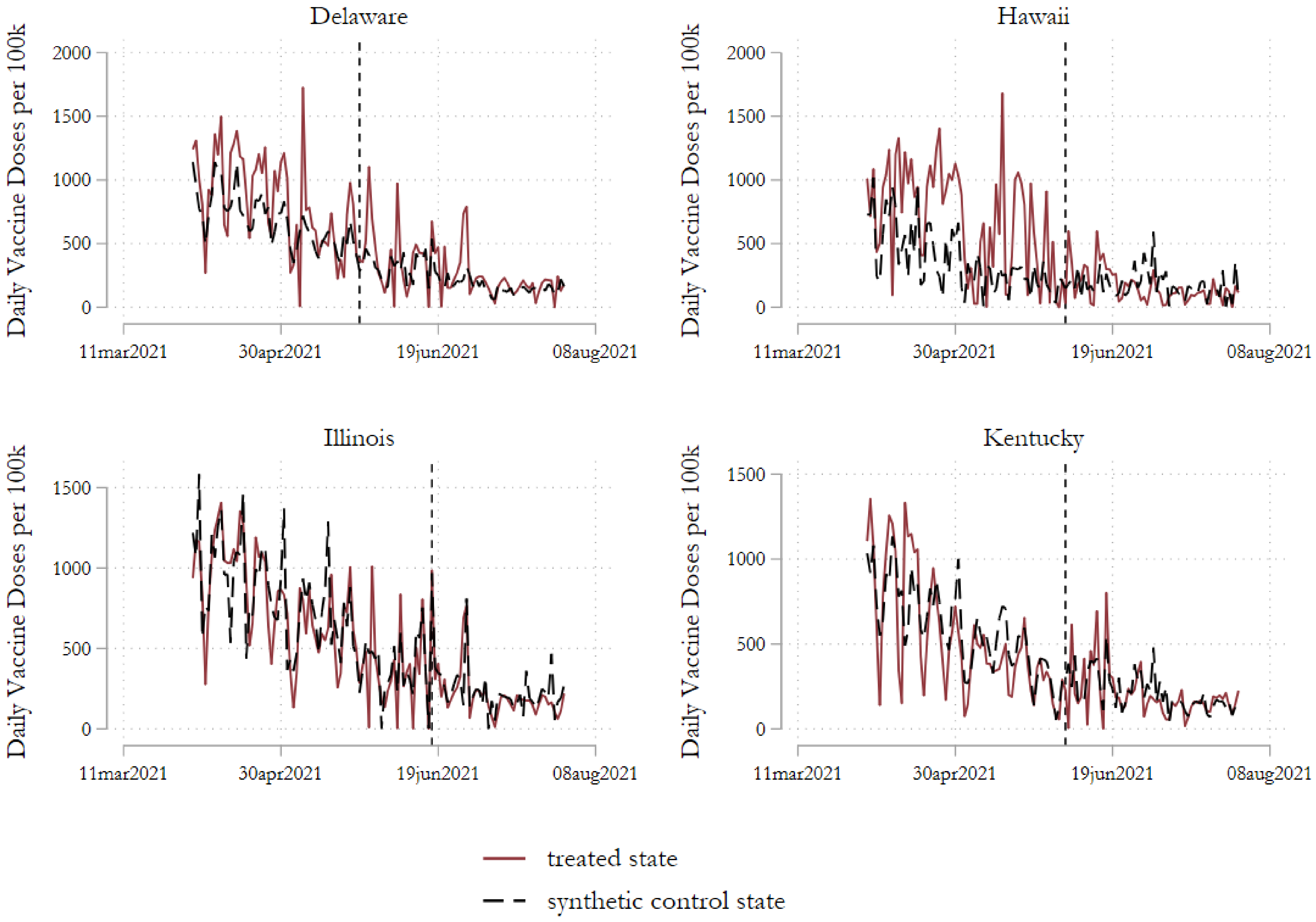

Delaware
Hawaii
Illinois
Kentucky
Daily Vaccine Doses per 100k
0
500
1000
1500
2000
11mar2021
30apr2021
19jun2021
08aug2021
treated state
synthetic control state

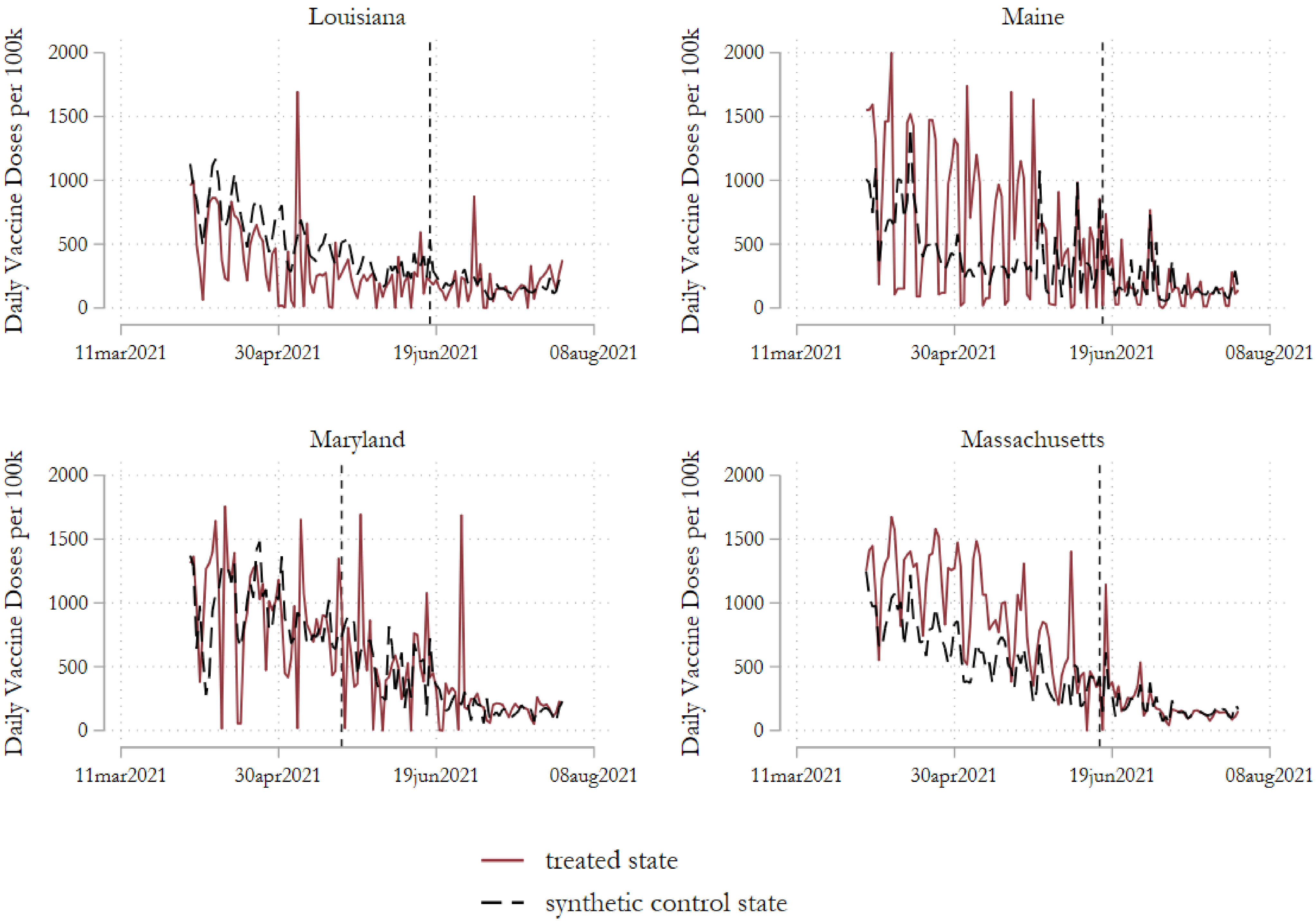
Louisiana
Maine
Maryland
Massachusetts
Daily Vaccine Doses per 100k
2000
1500
1000
500
0
11mar2021
30apr2021
19jun2021
08aug2021
treated state
synthetic control state

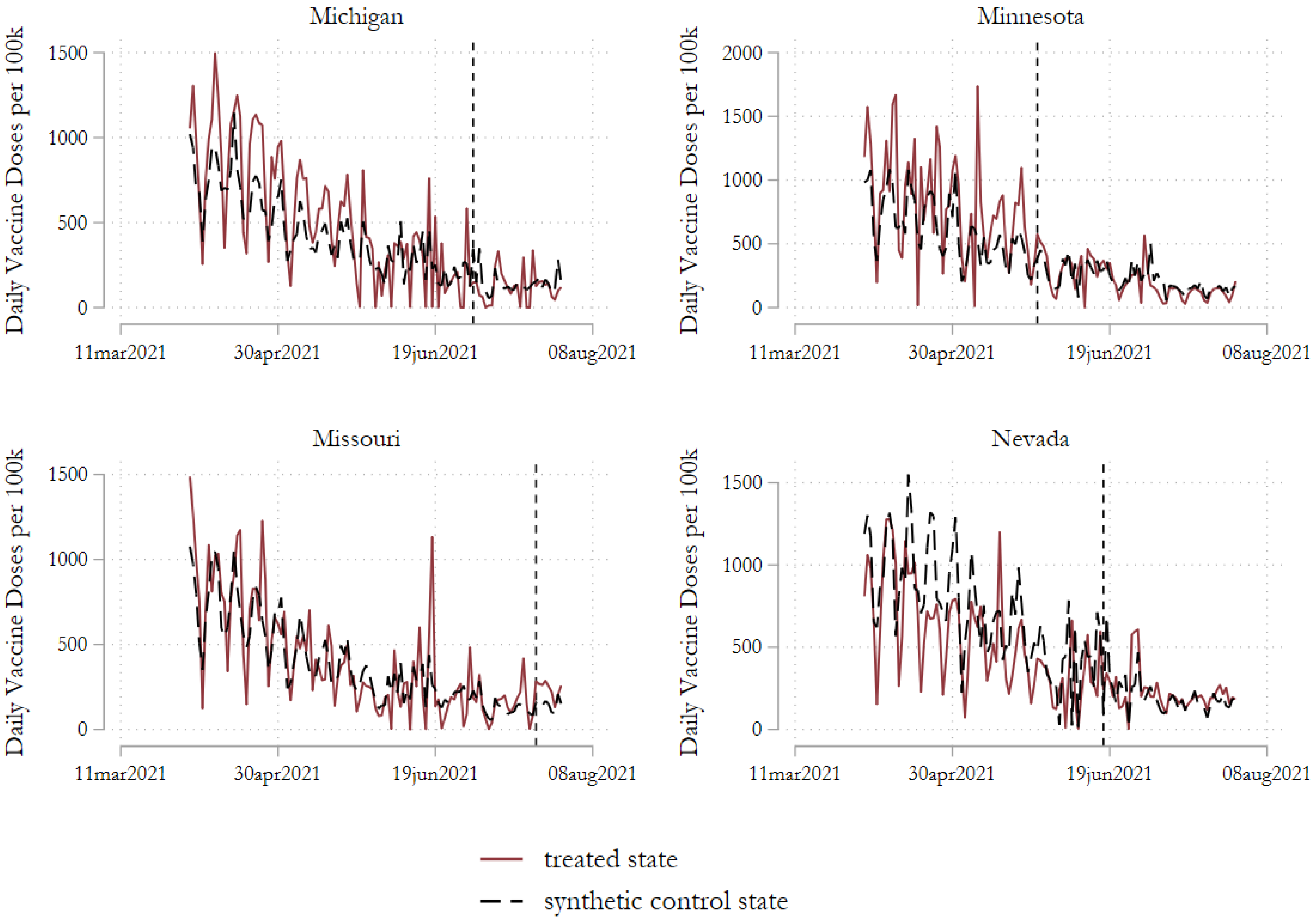

Michigan
Minnesota
Missouri
Nevada
Daily Vaccine Doses per 100k
0
500
1000
1500
2000
11mar2021
30apr2021
19jun2021
08aug2021
treated state
synthetic control state

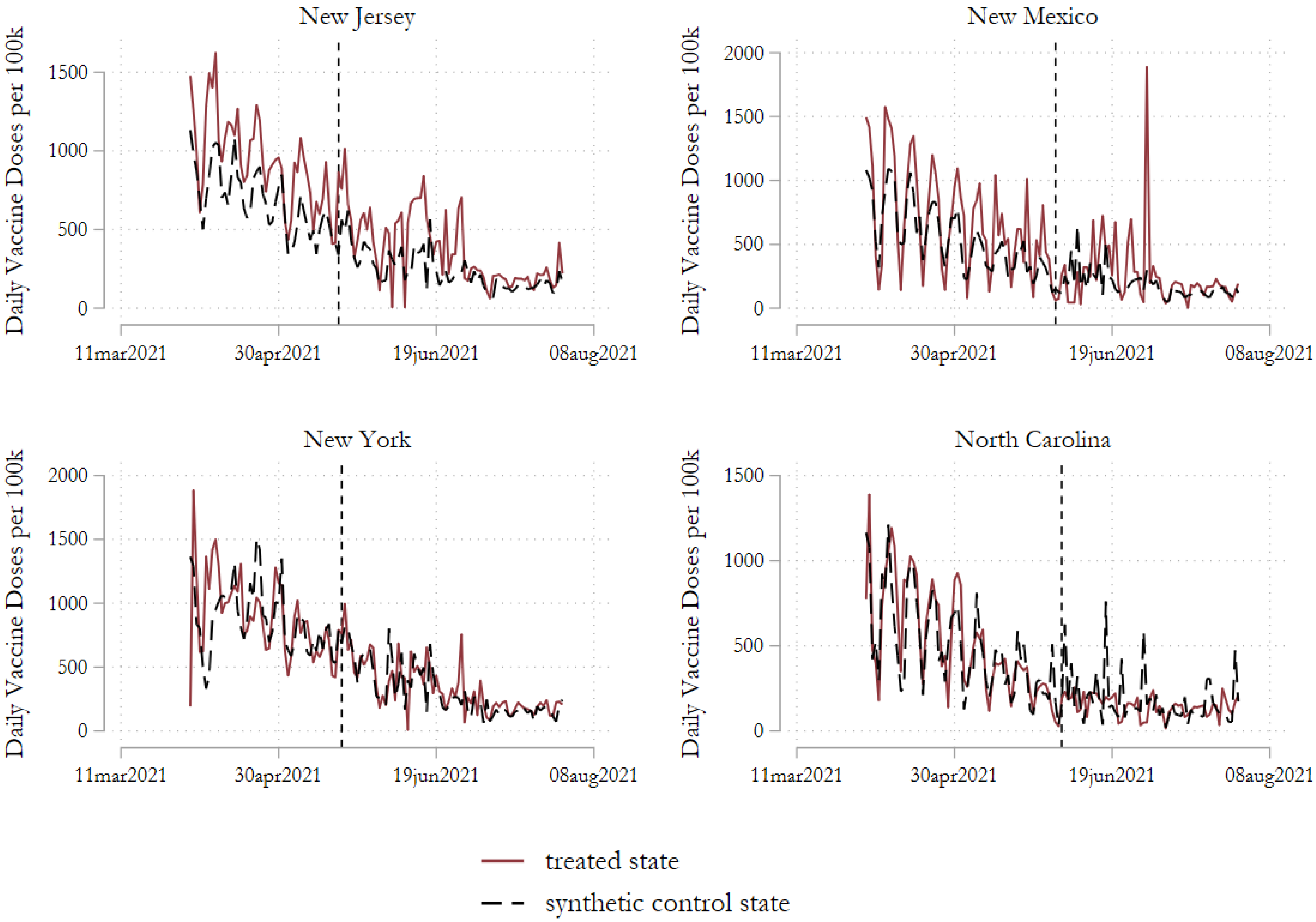
New Jersey
New Mexico
New York
North Carolina
Daily Vaccine Doses per 100k
0
500
1000
1500
2000
11mar2021
30apr2021
19jun2021
08aug2021
treated state
synthetic control state

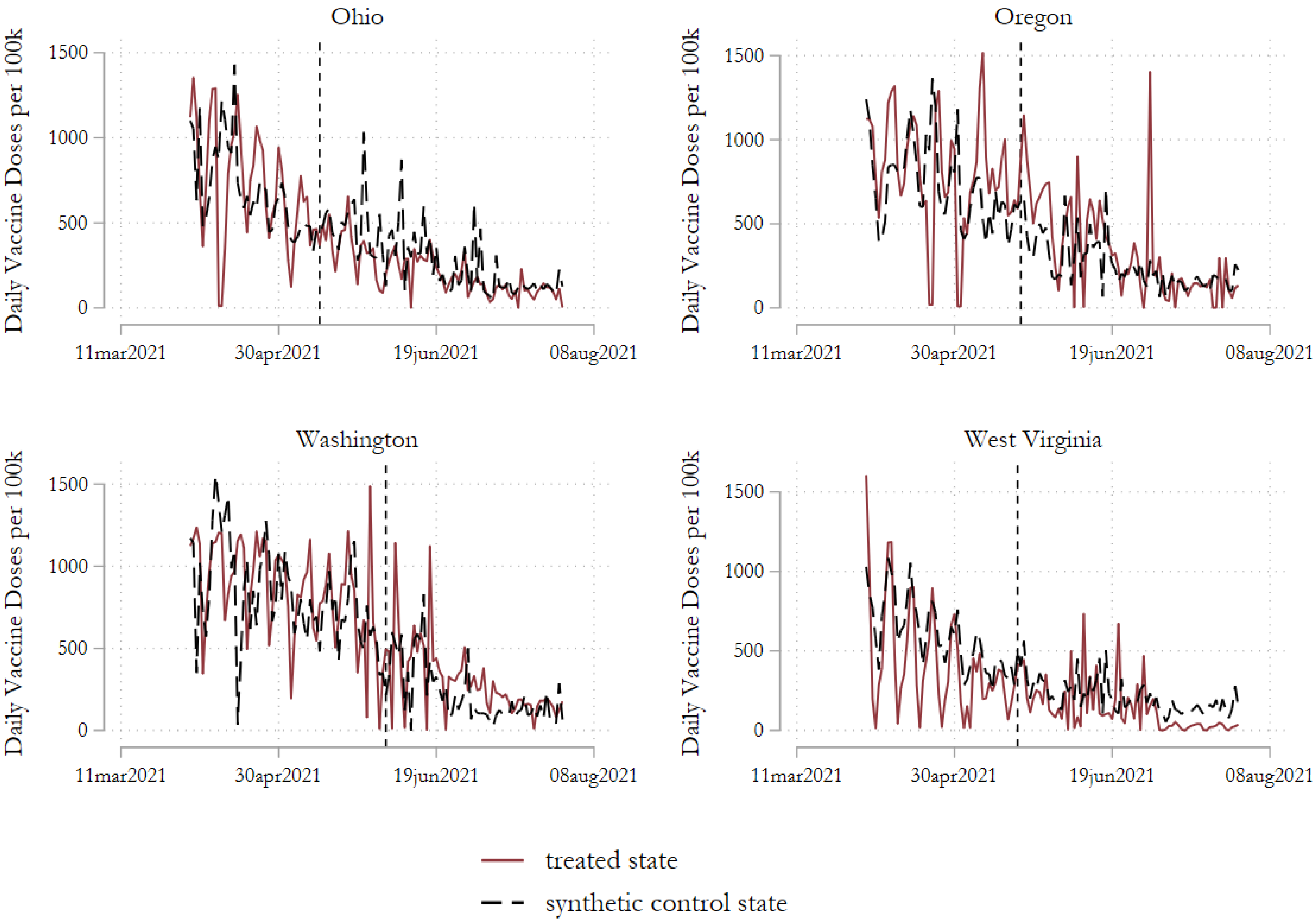
Ohio
Oregon
Washington
West Virginia
Daily Vaccine Doses per 100k
1500
1000
500
0
11mar2021
30apr2021
19jun2021
08aug2021
treated state
synthetic control state
